# Effects of Group Drumming Interventions on Anxiety, Depression, Social Resilience and Inflammatory Immune Response among Mental Health Service Users

**DOI:** 10.1371/journal.pone.0151136

**Published:** 2016-03-14

**Authors:** Daisy Fancourt, Rosie Perkins, Sara Ascenso, Livia A. Carvalho, Andrew Steptoe, Aaron Williamon

**Affiliations:** 1 Centre for Performance Science, Royal College of Music, Prince Consort Road, London, SW7 2BS, United Kingdom; 2 Faculty of Medicine, Imperial College London, South Kensington Campus, London, SW7 2AZ, United Kingdom; 3 Psychobiology Group, Department of Epidemiology and Public Health, University College London, 1–19 Torrington Place, London, WC1E 7HB, United Kingdom; University of Glasgow, UNITED KINGDOM

## Abstract

Growing numbers of mental health organizations are developing community music-making interventions for service users; however, to date there has been little research into their efficacy or mechanisms of effect. This study was an exploratory examination of whether 10 weeks of group drumming could improve depression, anxiety and social resilience among service users compared with a non-music control group (with participants allocated to group by geographical location.) Significant improvements were found in the drumming group but not the control group: by week 6 there were decreases in depression (-2.14 SE 0.50 CI -3.16 to -1.11) and increases in social resilience (7.69 SE 2.00 CI 3.60 to 11.78), and by week 10 these had further improved (depression: -3.41 SE 0.62 CI -4.68 to -2.15; social resilience: 10.59 SE 1.78 CI 6.94 to 14.24) alongside significant improvements in anxiety (-2.21 SE 0.50 CI -3.24 to -1.19) and mental wellbeing (6.14 SE 0.92 CI 4.25 to 8.04). All significant changes were maintained at 3 months follow-up. Furthermore, it is now recognised that many mental health conditions are characterised by underlying inflammatory immune responses. Consequently, participants in the drumming group also provided saliva samples to test for cortisol and the cytokines interleukin (IL) 4, IL6, IL17, tumour necrosis factor alpha (TNFα), and monocyte chemoattractant protein (MCP) 1. Across the 10 weeks there was a shift away from a pro-inflammatory towards an anti-inflammatory immune profile. Consequently, this study demonstrates the psychological benefits of group drumming and also suggests underlying biological effects, supporting its therapeutic potential for mental health.

***Trial Registration*:** ClinicalTrials.gov NCT01906892

## Introduction

Worldwide, mental health conditions are the leading cause of disability and, along with substance use disorders, are responsible for more of the global health burden than HIV/AIDS, tuberculosis, diabetes, or transport injuries. In the next 20 years, the global lost economic output as a result of mental health conditions will amount to $16 trillion [1].

The potential of music within mental health has been recognised for nearly a century [2,3], and there is now a large body of literature that has demonstrated improved symptoms and reduced severity of conditions, from depression to schizophrenia, in response to music [4,5]. However, the majority of previous studies have taken place within specific institutions and used a music therapy model, led by a professional music therapist with specific psychological aims. A much less researched area is whether *general* music making within community settings, not led by therapists, can still enhance the mental health and wellbeing of service users. This is an important topic to explore, as a growing number of organisations in the UK and abroad are developing community music interventions for mental health, including Youth Music UK and the Mental Health Foundation. Research into their efficacy is needed to ascertain whether they have a therapeutic effect and to support the design and implementation of future interventions.

One of the community music interventions growing in popularity for mental health is group drumming, perhaps due to the inclusiveness of drumming circles, lack of fine motor skill requirements and strong steadying rhythms. We conducted a preliminary study that explored whether group drumming sessions across six weeks could enhance indicators of mental health [6]. Our results showed significant improvements in standardized, self-report measures of depression, social resilience and mental wellbeing among participants. However, as the study was uncontrolled, it was not possible to rule out changes due merely to the passing of time or to a Hawthorne effect. The current study, therefore, involved a comparison of weekly 90-minute group drumming sessions with a control group who took part in other weekly social activities but were not actively involved in music interventions. As a midway point before conducting an RCT, in order to confirm previous findings and extend the intervention protocol, we designed this study as a controlled but non-randomised experiment. As such, this is an exploratory investigation into the effects of group drumming on mental health. We lengthened the duration of the drumming intervention from 6 to 10 weeks, with the aim of assessing in more detail the impact of this activity on mental health. We hypothesized that group drumming would lead to reductions in reported anxiety, depression, and stress alongside improvements in mental wellbeing and social resilience, with no systematic changes in the control group. We hypothesized that these changes in the drumming group would be maintained 3 months following the end of the intervention.

Furthermore, research over the past two decades has demonstrated that many mental health conditions are characterized by an underlying imbalance between pro- and anti-inflammatory cytokines (proteins within the immune system), weighted towards a pro-inflammatory response. Pro-inflammatory cytokines can act as neuromodulators, orchestrating behavioural changes associated with depression [7,8]. Successful management of mental health conditions, whether through pharmacotherapy, psychotherapy or even psychosocial programmes such as reminiscence session and social activities, has been shown to correspond with decreases in pro-inflammatory cytokines and a rebalancing of the pro- versus anti-inflammatory ratio [9–11]. There is a growing body of evidence that music can lead to immune-modulation [12]. However, there are limited data on whether music can exert the same pro- vs anti-inflammatory rebalancing in mental health service users. So, this study also included exploratory analyses of immune function, with the hypothesis that, alongside improvements in wellbeing, drumming would be associated with a reduction in pro-inflammatory immune response and a shift towards an anti-inflammatory immune profile across the 10 week intervention and a reduction in the stress hormone cortisol [6,12].

## Materials and Methods

### Design and participants

This was a parallel group comparison of 10 weeks of drumming versus a control group who engaged in regular community-based (non-musical) social activities, with a 3-month follow-up of the drumming condition. Fifty-nine participants were recruited to the two groups of whom forty-five completed the study: 30 experimental and 15 controls. All participants were adults accessing mental health services and were recruited from hospitals, through psychologists and psychiatrists working in the National Health Service (NHS) or private practice; or through professional mental health support or carer organizations and charities. All participants self-selected to take part and contacted the study team directly. Experimental participants were recruited from West London near to the location of the intervention for ease of access. Control participants were recruited through the same channels in South and North London. To minimise potential bias, they met the same inclusion criteria but were not within the vicinity of the group drumming sessions to take part. The groups were matched for age, sex, ethnicity and employment status, within the constraints of our recruitment channels (see Table 1). Drumming participants were not blinded as to which study condition they were in. Control participants were told they were participating in a study about music and mental health but were not aware that they could have had access to a drumming group had they lived in West London. Staff collecting saliva samples were not blinded, but laboratory analysis was blind.

**Table 1:** Baseline data.

	Drumming (n = 30)	Control (n = 15)	Difference
**Female, *n* (%)**	23 (77%)	14 (93%)	p = 0.169^a^
**Age, mean ± SD**	55.07 ± 13.0	52.00 ± 14.7	χ^2^_44_ = 0.51, p = 0.480
**Ethnicity white, *n* (%)**	21 (70%)	15 (100%)	χ^2^_3_ = 5.63, p = 0.131
**Status, *n* (%)**			χ^2^_3_ = 7.19, p = 0.066
** Employed**	4 (13%)	7 (47%)	
** Retired**	10 (33%)	4 (27%)	
** Other**	16 (53%)	4 (27%)	
**Education**			χ^2^_3_ = 0.47, p = 0.925
** No formal qualifications**	4 (13%)	1 (7%)	
** GCSE/A2**	7 (23%)	4 (27%)	
** Undergraduate qualification**	13 (43%)	7 (47%)	
** Postgraduate qualification**	6 (20%)	3 (20%)	
**Musical experience, *n* (%)**			
** Currently play an instrument**	11 (37%)	11 (13%)	p = 0.162^a^
** Previously play an instrument**	13 (43%)	8 (53%)	p = 0.755^a^
** Previous experience of drumming**	0	0	p = 1.00^a^
** Regularly listen to drumming**	0	3 (10%)	p = 0.540^a^
**Psychological scales, mean ± SEM**			
** HADSA**	11.03 ± 0.83	9.93 ± 1.16	F_1,42_ = 0.591, p = 0.446
** HADSD**	8.90 ± 0.79	4.27 ± 1.10	F_1,42_ = 11.625, p = 0.001
** CDRISC**	46.93 ± 3.47	57.85 ± 4.83	F_1,42_ = 3.385, p = 0.073
** WEMWBS**	39.61 ± 1.91	44.67 ± 2.61	F_1,42_ = 0.355, p = 0.555
** PSS**	23.17 ± 1.28	21.87 ± 1.78	F_1,40_ = 3.554, p = 0.125

Baseline demographics and psychological profile for the drumming and control groups.

^a^ Fisher’s exact test

Participants were excluded if they had a dementia that prevented them from giving informed consent, or a severe hearing or physical impairment that would have prevented them from taking part in the drumming intervention. Patients continued with their usual care during the intervention, but if there was a change to their care over the 10 weeks, such as commencing a new or different therapy, a change in their medication or a new diagnosis, they were retrospectively excluded from the analysis. Participants were further excluded from biological analyses if they were taking anti-depressants, steroids or anti-anxiety medication or if they had a gum disease that may cause local inflammation in the mouth (n = 3). This, along with some samples falling below the limits of detection during laboratory analysis, accounts for variations in the degrees of freedom reported. The study protocol (S1 Protocol) was approved by the UK NHS National Research Ethics Service, and all participants gave written informed consent prior to the study.

### Procedure

Experimental participants took part in weekly 90-minute group drumming sessions over a period of 10 weeks. They were split into two groups of 15–20, and sessions ran consecutively on weekday mornings in a hired community space in West London, led by a professional drummer with experience leading community music activities and supported by three students from the Royal College of Music. Each participant was provided with a djembe drum and sat in a circle. The professional drummer taught the participants the basics of how to use the drum, led the participants in a series of ‘call-and-response’ exercises where participants copied the leader, and taught the participants rhythmic patterns that were eventually employed over the six weeks in a larger drumming piece. Participants also had small sections of free improvisatory drumming creating musical accompaniment to different scenarios such as the sound of water. The professional drummer gradually increased the complexity of the drumming as the weeks went on but moved at the pace of the participants, waiting until the group was comfortable before adding more difficult elements. Talking took up an estimated 20% of the session time, as the leader explained new material to the participants, but the general teaching approach was by demonstration rather than didactic. Unlike music therapy, the professional drummer was not told details of participants’ backgrounds or psychological profiles and did not have specific psychological or therapeutic aims. His brief was rather to lead a community drumming session that suited the abilities of the participants and then teach them new techniques and rhythms to expand their drumming skills. Participants had to attend a minimum of 8 out of 10 sessions to be included in the analysis. Control participants did not take part in any group musical activities during the 10 weeks. However, to reduce the bias of having control subjects who were merely more socially isolated than the experimental group, all control subjects were already regular participants in community group social activities (e.g. quiz nights, women’s institute meetings and book clubs) and continued with these activities for the duration of the study. No incentives were given to participants.

### Psychological measures

Participants’ socio-demographic and mental health characteristics were obtained by means of a set of self-administered questionnaires. Our primary outcome measure was anxiety and depression, which we measured using the Hospital Anxiety and Depression Scale (HADS) [13], ranging from 0–21 for each construct with higher scores indicating poorer mental health. For our secondary outcome measures, we assessed wellbeing using the Warwick-Edinburgh Mental Wellbeing Scale (WEMWBS), a questionnaire used extensively as a measure of mental health ranging from 14–70 with higher scores indicating better wellbeing [14]; stress with the Perceived Stress Scale [15], which ranges from 0–40 with higher scores indicating higher stress; and social function with the Connor-Davidson Resilience Scale (CDRISC) ranging from 0–100 with higher scores indicating stronger resilience [16].

### Biological measures

Given recent research suggesting the promise of saliva as an alternative to blood analysis in biobehavioural research [17–20], this study used the technique of multiplex saliva assays for the assessment of cortisol and cytokines. As this was an exploratory analysis, biological measures were obtained only from participants in the drumming condition. Saliva was collected via a passive drool method facilitated by polypropylene straws into low-bind polypropylene 2mL cryovials (Eppendorf, UK). Sampling took place for all participants between 10am and 12noon with each participant providing their sample at the same time at each data collection point. Samples were stored at -20°C for a period of 2–8 weeks before transfer to -40°C for one month prior to analysis at Aeirtec Laboratories. Samples were vortexed and centrifuged before analysis. Supernatants were pipetted into 96 well filter plates (Millipore, UK) with microplex_TM_ beads (Luminex Corp., USA) conjugated with antibodies to the analytes.

The samples were analyzed for the neuroendocrine stress hormone cortisol, and immune cytokines interleukin (IL) 4, IL6, IL17, tumour necrosis factor alpha (TNFα), and monocyte chemoattractant protein (MCP) 1. Interleukin 4 is a well-established anti-inflammatory marker [21], while TNFα is a pro-inflammatory marker, providing representatives from both groups [22]. IL6 and IL17 have both pro- and anti-inflammatory properties and have been shown to be sensitive to change in mental health service users in previous studies [23,24]. MCP1 is a further pro-inflammatory marker that has also been found to reduce in response to pharmacological treatment for mental health conditions [25].

All capture and detection antibodies and standards were purchased from Perotech, UK, with the exception of TNF-α (R&D Systems, UK) and cortisol (antibodies from Abcam, UK, tracer from Randox, UK, and standards from Sigma-Aldrich, UK). Following incubation for 24 hours, the beads were washed by filtration and then biotin-conjugated antibodies to the analytes were added to the beads for 4 hours. Following incubation with detection antibodies, the beads were washed and incubated with streptavidin-conjugated R-phycoerythrin for 45 minutes to provide a fluorescent detection signal. Following washing, the beads were analysed on a Luminex 100_TM_ analyser. The inter-assay coefficient of variation range for all analytes was 1.8–5.37%, and the intra-assay coefficient of variation range was 0.8–3.58%.

### Statistical analyses

Sample size was calculated using data from the previous six-week study with the primary endpoint of depression (HADSD) which showed an effect size of f = 0.6 [6]. Using this effect size, an a priori sample size calculation using G*Power 3.1 for a between-factors ANOVA with an alpha of 0.05, power of 0.9 and assuming two-sided tests and a correlation of 0.8 among repeated measures (2 groups, 3 timepoints) was made which showed that an overall total of 28 participants would be required (14 per group). For the control group, to allow for drop-outs of 30% (estimated based on the six-week study), 20 participants were targeted for recruitment. For the experimental group, because of the range of biological markers being tested, we decided to match sample size with our preliminary study [6], and so 39 participants were initially recruited. Recruitment was continued until these targets had been reached before being closed one week before the drumming started. Following drop-outs, 15 control and 30 drumming participants remained.

Data were analysed using IBM SPSS version 21.0.1 (SPSS, Chicago, IL). Data were analysed at a group level. For psychological data, baseline scores between the two groups were compared using one-way analyses of variance (ANOVAs; see Table 1). Differences between groups in baseline demographic data were compared using Chi-squared test and Fisher’s exact test. For psychological data, we used 3 x 2 repeated measures ANOVAs to examine within-subjects effects of time (baseline, week 6, week 10), between-subjects effects of condition (drumming vs control groups), and their interaction. Based on data from the preliminary study, linear effects were assumed so we used planned simple contrasts (baseline vs week 10, week 6 vs week 10) to assess within-subjects changes. Due to a difference in the baseline scores between the drumming and control groups in depression (HADSD) (see Table 1), these data were also analyzed with a univariate analysis of covariance (ANCOVA) comparing the change scores between groups from baseline to week 6 and baseline to week 10, with the baseline values as a covariate in order to confirm whether change across time was significant when controlling for them.

For the follow-up psychological data, which were collected 3 months after the end of the intervention only for the drumming group, we used two repeated measures ANOVAs with within-subjects effects of time: one ANOVA to assess whether final psychological levels were significantly different from the start of the intervention (baseline vs 3 months follow-up); and one ANOVA to assess whether final psychological levels were significantly different from the end of the intervention (week 10 vs 3 months follow-up).

For biological data, we again used repeated measures ANOVAs with time (baseline, week 6, week 10) as the within-subjects factor. The distribution of all cytokines was positively skewed, so data were logarithmically transformed prior to analysis. Given that cytokines can show rebound effects across time, we used planned polynomial contrasts to assess whether biomarkers showed linear or quadratic trends. In order to calculate TNFα vs IL4 response as markers of pro-inflammatory and anti-inflammatory profiles respectively, z scores for both biomarkers were created and comparisons between them were made at baseline, week 6, and week 10 using t-tests.

For all ANOVAs, data were continuous and normally distributed (or log-transformed to achieve a normal distribution for cytokines), with no significant outliers and evidence of sphericity from Mauchley’s test.

## Results

**Fig 1:**
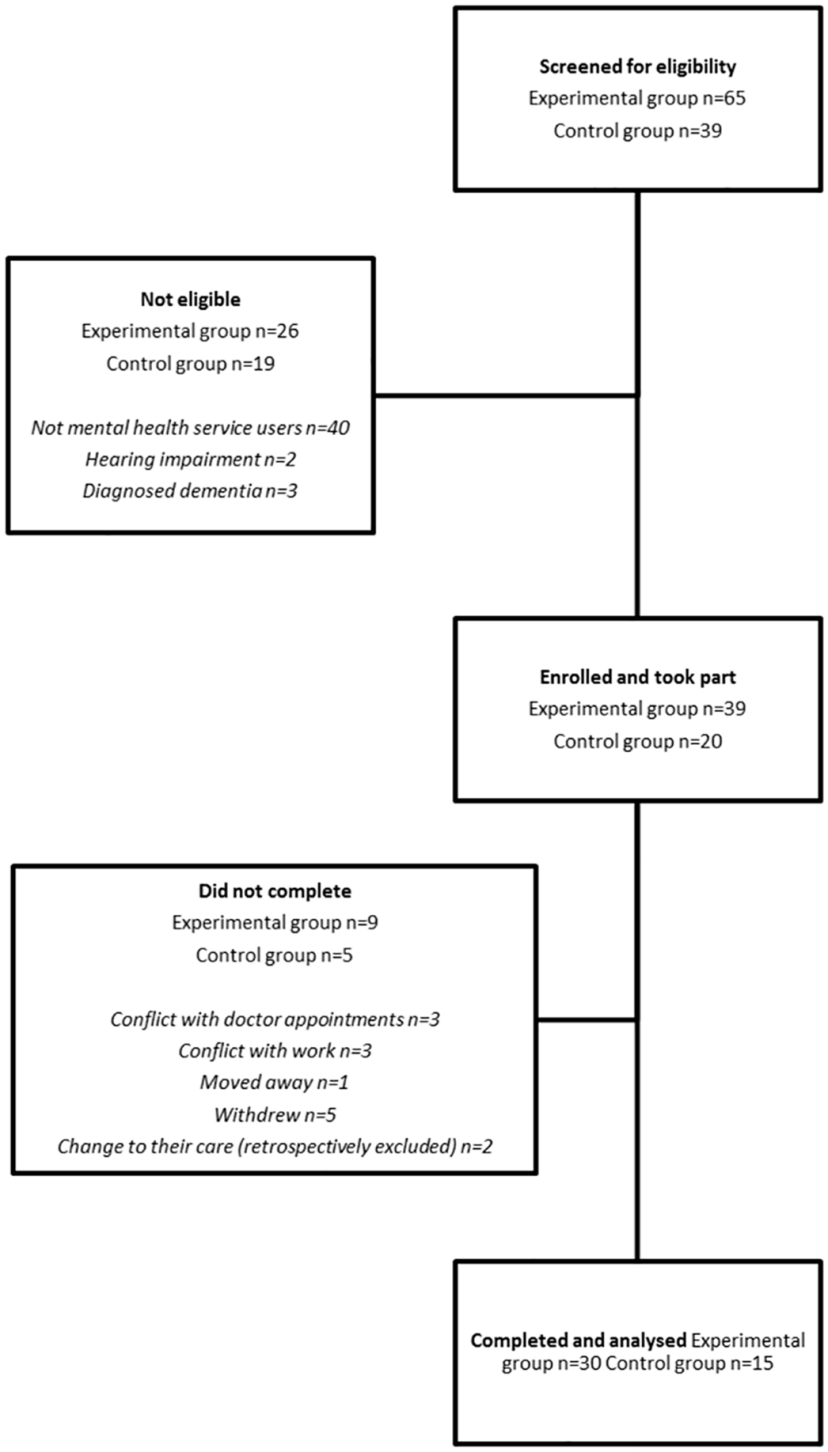
Flow of participants involved in the study.

One hundred and four participants were screened for eligibility and fifty-nine enrolled into two groups (Fig 1). Where a full data set was missing from one of the 3 timepoints in the study due to non-attendance or dropping out, participants were excluded from analyses (n = 9). Where data had been collected at all timepoints, but a single piece of data was missing, such as due to a participant forgetting to answer a question, participants were included and degrees of freedom for that particular measure adjusted accordingly. A total of 30 experimental and 15 control participants completed participation and were analysed. Exploratory analyses confirmed that there were no differences in baseline measures between participants who completed the study and participants who dropped out. Participants were recruited from August to October 2014 and undertook study participation from October to December 2014. There were no deviations from the study protocol nor adverse events.

The drumming and control groups were well matched at baseline across all psychological measures except for depression, where the drumming group reported significantly higher levels (see Table 1). There were also no demographic differences between participants included in the final analysis and participants who withdrew from the study.

Participants displayed moderate anxiety on the Hospital Anxiety and Depression Scale (HADSA: 10–15); mild depression in the drumming group on the Hospital Anxiety and Depression Scale (HADSD: 8–10) [26]; social resilience scores below average adults (CDRISC: 80.7) and scores below those of generalized anxiety patients on the Connor-Davidson Social Resilience Scale (CDRISC: 62.4) [16]; mental wellbeing scores below the UK national average on the Warwick-Edinburgh Mental Wellbeing scale (WEMWBS: 51.6) [27]; and stress scores higher than average on the Perceived Stress Scale (PSS: 13), indicative of high stress (PSS: >20) [15].

### Psychological results

Participants completed the psychological scales at baseline (week 1) and weeks 6 and 10. Full descriptive statistics for both conditions are provided in S1 Table.

**Fig 2:**
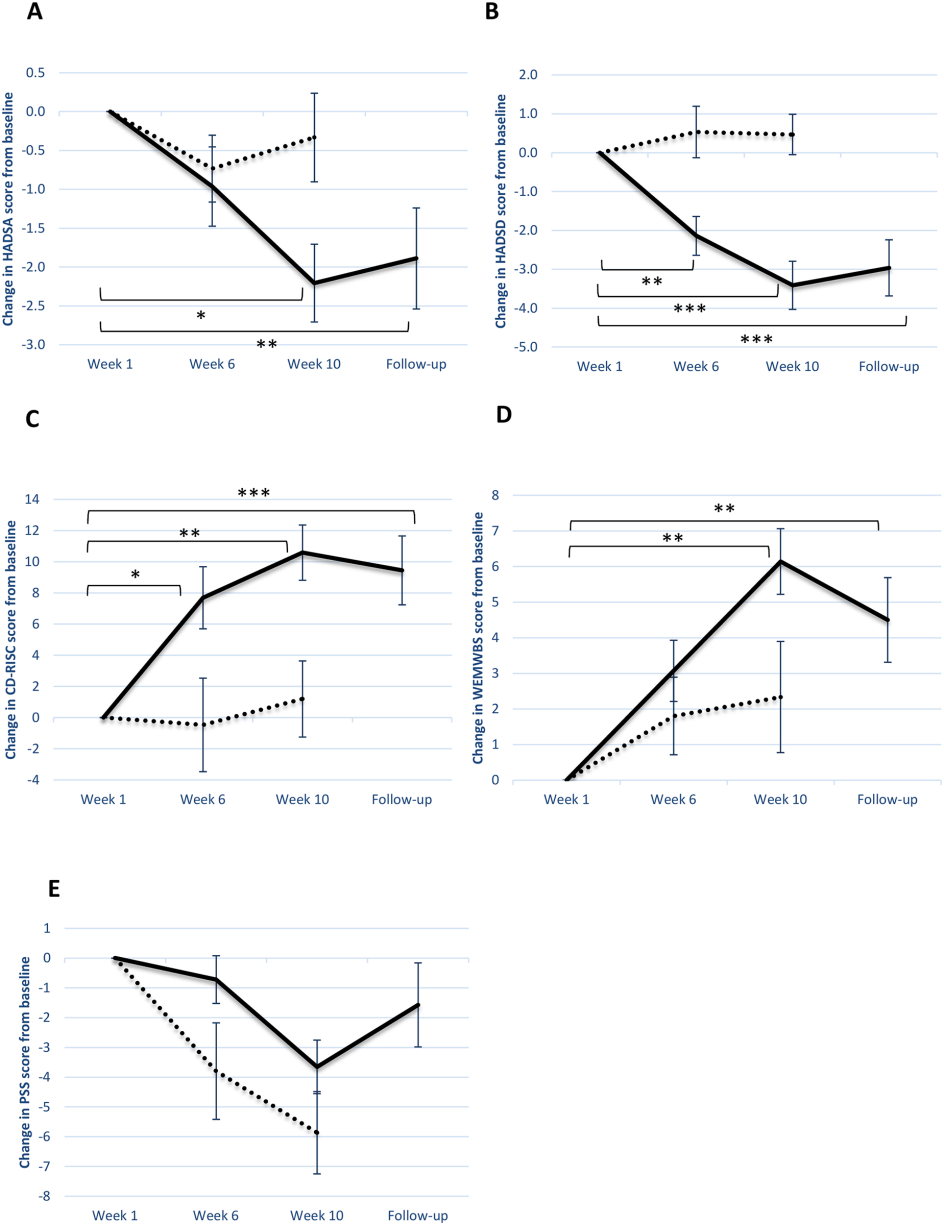
Psychological results. Within-subject change from baseline (with standard error) at weeks 6 and 10 for drumming and control groups for (A) Anxiety (HADSA), (B) Depression (HADSD)(C) Social Resilience (CDRISC), (D) Mental Wellbeing (WEMWBS) (E) and Perceived Stress (PSS). * = p < .05, ** = p < .01, *** = p < .001.

The analysis of anxiety ratings showed a significant condition by time interaction (F_2,84_ = 3.63, p<0.05), with anxiety falling over the 10 weeks of drumming (mean -2.21, SE 0.50, CI -3.24 to -1.19) while remaining unchanged in the control condition (mean -0.33, SE 0.57, CI -1.55 to 0.88). Within-subjects contrasts showed that the time by group interaction did not reach significance at 6 weeks but did reach significance by 10 weeks (F_1,42_ = 5.357, p<0.05). The overall decrease in anxiety from baseline in the drumming group averaged 9% by week 6 and 20% by week 10. Fig 2A shows the within-subject change from baseline at weeks 6 and 10 in both the drumming and control conditions.

The analysis of depression ratings showed a similar pattern, with a significant condition by time interaction (F_2,84_ = 10.23, p<0.001). Depression fell over the 10 weeks of drumming (mean -3.41, SE 0.62, CI -4.68 to -2.15) while remaining unchanged in the control condition (mean 0.47, SE 0.52, CI -0.66 to 1.59). Within-subjects contrasts showed that the time by group interaction reached significance at 6 weeks (F_1,42_ = 10.038, p<0.01) and was seen even more strongly by 10 weeks (F_1,42_ = 17.048, p<0.001). The overall decrease in depression from baseline in the drumming group averaged 24% by week 6 and 38% by week 10. In the light of the baseline differences in depression ratings, we also analyzed change scores over time controlling for baseline levels; the difference between drumming and control conditions remained significant at week 10 (F_1,41_ = 5.035, p<0.05). Fig 2B shows the within-subject change from baseline at weeks 6 and 10 in both the drumming and control conditions.

There was also a significant condition by time interaction for social resilience (F_2,84_ = 5.13, p<0.01), with improvements across 10 weeks in the drumming group (mean 10.59, SE 1.78, CI 6.94 to 14.24) but not the control group (mean 1.20, SE 2.44, CI -4.03 to 6.43). Within-subjects contrasts showed that the time by group interaction reached significance at 6 weeks (F_1,42_ = 5.393, p<0.05) and was even stronger by 10 weeks (F_1,42_ = 9.563, p<0.01). The overall improvement in social resilience in the drumming group averaged 16% by week 6 and 23% by week 10. Fig 1C shows the within-subject change from baseline at weeks 6 and 10 in both the drumming and control conditions. Fig 2C shows the within-subject change from baseline at weeks 6 and 10 in both the drumming and control conditions.

In the analysis of the wellbeing scores, the condition by time interaction was nearly significant (F_2,82_ = 2.91, p = 0.06), with changes in the drumming group (mean 6.14, SE 0.92, CI 4.25 to 8.04) but not the control group (mean 2.33, SE 1.56, CI -1.01 to 5.67). Within-subjects contrasts showed that the time by group interaction was not significant at 6 weeks but was significant by 10 weeks (F_1,41_ = 5.033, p<0.05). The improvement in the drumming group averaged 8% by week 6 and 16% by week 10. Fig 2D shows the within-subject change from baseline at weeks 6 and 10 in both the drumming and control conditions.

The analysis of perceived stress showed a significant main effect of time (F_2,84_ = 18.77, p<0.001) but no condition by time interaction (p = 0.131); perceived stress fell over time in both groups (drumming: mean -3.66, SE 0.90, CI -5.49 to -1.82; control: mean -5.87, SE 1.38, CI -8.83 to -2.90). Fig 2E shows the within-subject change from baseline at weeks 6 and 10 in both the drumming and control conditions.

### Follow-up psychological results

In order to evaluate whether the effect of group drumming on the psychological results was maintained after the intervention, the scales were administered with the drumming group at 3 months follow-up and compared statistically with baseline levels. The observed changes were maintained, with significant differences at follow-up from baseline (HADSA: F_1,26_ = 8.386, p<0.01; HADSD: F_1,26_ = 16.703, p<0.001; CDRISC: F_1,26_ = 18.157, p<0.001; WEMWBS: F_1,25_ = 14.299, p<0.01) (Fig 2). We also compared whether these follow-up levels had changed since the end of the intervention (week 10), finding no significant differences (HADSA: F_1,26_ = 0.137, p = 0.715; HADSD: F_1,26_ = 0.273, p = 0.606; CDRISC: F_1,26_ = 0.795, p = 0.381; WEMWBS: F_1,25_ = 1.894, p = 0.181).

### Biological results

**Fig 3:**
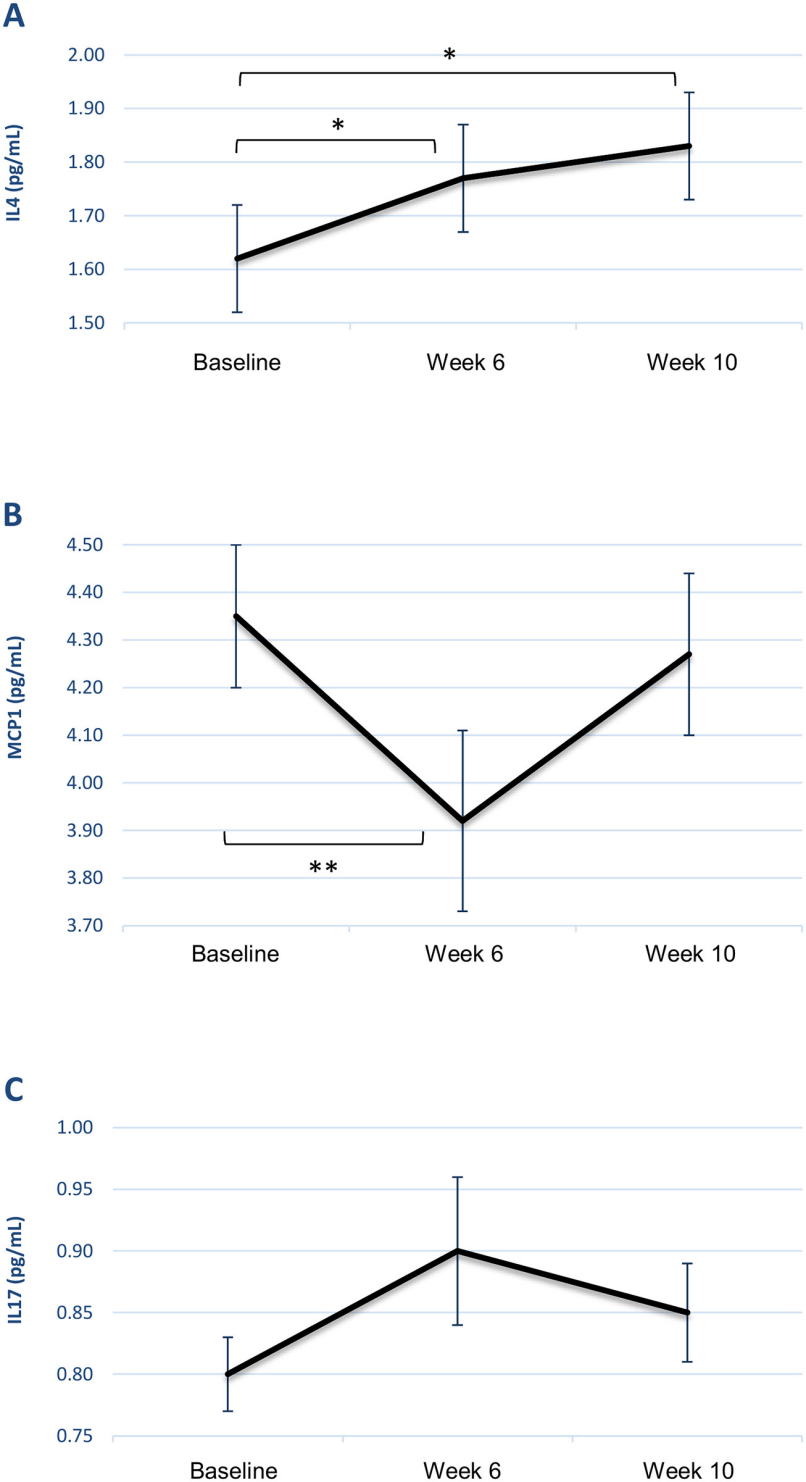
Biological results. Mean cytokines levels (with standard errors) in response to drumming across the 10 weeks for(A) interleukin 4 (IL4), (B) monocyte chemoattractant protein 1 (MCP1), and (C) interleukin 17 (IL17). * = p < .05, ** = p < .01.

In order to explore the mechanisms of change in the drumming group, exploratory saliva samples were taken immediately before and after baseline and weeks 6 and 10 of drumming. Drumming significantly increased the anti-inflammatory cytokine IL4 (F_2,34_ = 3.830, p<0.05). Planned polynomial contrasts showed that there was a linear effect across time (F_1,17_ = 6.504, p<0.05), with a 9% increase in levels by week 6 and a 13% increase by week 10 (see Fig 3A). Alongside this, there was a significant change in levels of the pro-inflammatory chemokine MCP1 (F_2,30_ = 4.221, p<0.05), which polynomial contrasts revealed to be a quadratic effect, with a decrease of 10% by week 6 followed by a return to near baseline levels by week 10 (F_1,15_ = 10.793, p<0.01) (see Fig 3B). There was also a near-significant effect for IL17 (F_2,30_ = 2.502, p = 0.099), also shown to be a quadratic effect characterized by an initial increase of 13% followed by a small decrease returning to 6% above baseline levels (F_1,15_ = 4.301, p = 0.056) (see Fig 3C). No changes were found in levels of TNFα across the 10 weeks (F_2,34_ = 0.134, p = 0.875) nor IL6 (F_2,34_ = 0.808, p = 0.454), and although there was a decrease in cortisol across the 10 weeks, this was not significant (F_2,42_ = 0.593, p = 0.557). Full descriptive statistics are provided in S2 Table.

**Fig 4:**
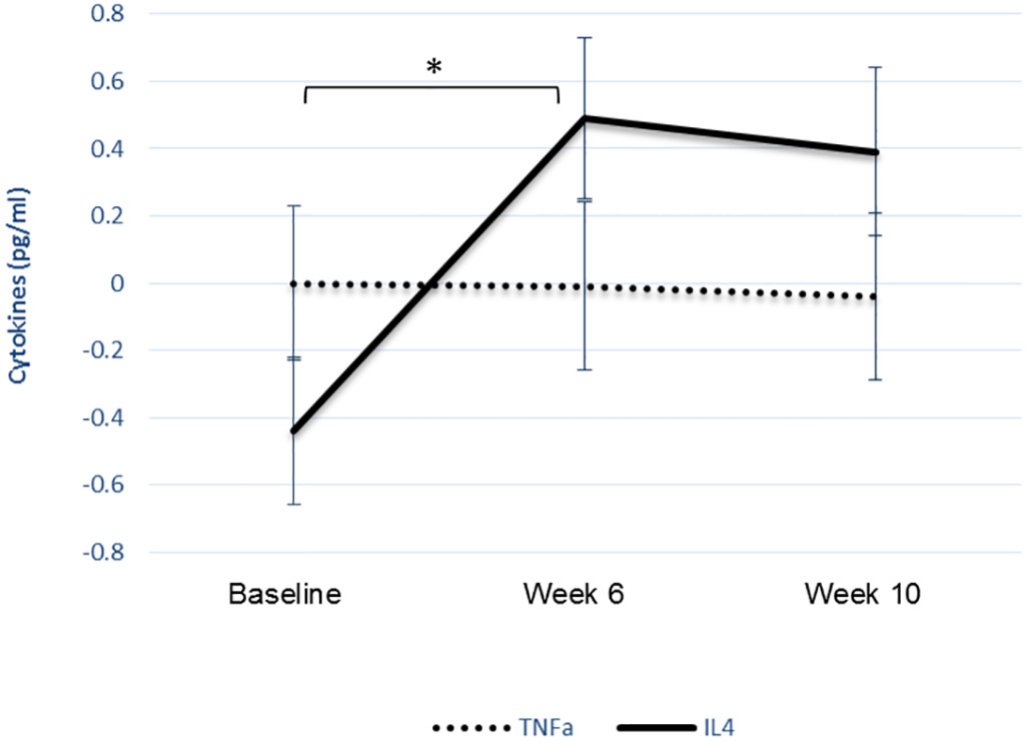
Pro- vs anti-inflammatory responses. Mean z scores for TNFα and IL4 (with standard errors) in response to drumming across the 10 weeks.

A further comparison of pro- versus anti-inflammatory response was calculated by comparing levels of the cytokines TNFα (pro-inflammatory) and IL4 (anti-inflammatory) with standardized scores, a technique used in previous research exploring psychobiological responses in mental health [28]. At baseline, a comparison of levels of TNFα and IL4 was elevated towards TNFα (a pro-inflammatory response); however, over the intervention period, there was a shift towards IL4 (an anti-inflammatory response) which reached significance by week 6 (t_16_ = -2.193, p<0.05) (see Fig 4).

### Psychobiological interactions

At baseline, there were no significant correlations between psychological and biological measures. There were also no significant correlations between baseline psychological levels and changes in biomarkers. However, there was a positive correlation between baseline IL17 levels and changes in wellbeing, with higher baseline IL17 associated with greater improvements in wellbeing (r = .469, p = .028). No other correlations between baseline biological levels and psychological changes were found.

When comparing changes in psychological measures with changes in biological measures, in the drumming group, there was a negative correlation between changes in IL4 and changes in anxiety across the 10 weeks, with decreases in anxiety associated with increases in IL4 (r = -.398, p = .044) and a near-significant negative correlation between changes in IL17 and changes in social resilience (r = -.381, p = .080).

## Discussion

This study explored whether a music-making intervention, specifically group drumming, could improve mental health over several weeks and whether psychological responses were found in parallel with a reduction in pro-inflammatory response in order to ascertain the in-depth effects of drumming on mental health service users.

We hypothesized that across the ten-week intervention there would be a decrease in symptoms of depression and anxiety and improvements in social resilience and mental wellbeing. This was demonstrated by significant reductions in HADSA and HADSD and significant increases in CDRISC and near-significant improvements in WEMWBS, compared with the control group. We also hypothesized that the changes seen in the drumming group would be maintained at 3-month follow-up, which was also confirmed. Furthermore, for the drumming group, we tested whether there would be a decrease in pro-inflammatory cytokine activity; the results showed a significant increase in the anti-inflammatory cytokine IL4 and a shift towards an anti-inflammatory profile, as shown by the balance between levels of TNFα and IL4.

This study demonstrates that group drumming leads to enhanced psychological states, specifically less depression and greater social resilience, across six weeks compared with a control group, extending the findings of our preliminary study [6]. Although in both the preliminary and the current study the decrease in anxiety was not significant across the first six weeks, this study demonstrated that, when the drumming was extended to 10 weeks, the decrease was significant. It is of note that between weeks 6 and 10 the improvements in psychological states continued, suggesting that the 10-week intervention was more effective. These findings are comparable with findings from previous music studies involving anxiety and depression, such as Coulton et al. (2015) who also found significant improvements across 3 months of group singing in older adults and Erkkila et al. (2011) who found significant improvements across 3 months of individualised music therapy with patients with depression [4,29]. However, both studies found that results returned to near baseline levels (producing non-significant results compared with baseline) at follow-up 3 months after the end of the music intervention. In contrast, this study showed that results were maintained for 3 months after the end of the intervention. It remains unknown whether even longer interventions could further improve outcomes; however, given funding limitations for community interventions [30], it is promising that just 10 weeks have been shown to lead to results being maintained for three months after the end of the intervention.

A second finding of the study was that exploratory biological analyses revealed that drumming led to increases in anti-inflammatory activity. The intervention led to increases in the anti-inflammatory cytokine IL4, both between baseline and week 6 and continuing to week 10. When levels of TNFα and IL4 were assessed, there was a shift away from a pro-inflammatory profile towards an anti-inflammatory response. The changes noted in these cytokines are relatively small so future research remains to be carried out as to their biological significance. However, they are in line with theories of Dahl et al. (2014) who suggested that measurable improvements in mental health occur alongside shifts towards an anti-inflammatory response. It also supports and extends findings from our preliminary study [6]. Furthermore, as TNFα and IL4 are also markers of Th1 and Th2 immune pathways respectively, this study suggests a shift away from Th1 responses in favour of Th2 responses, which is in line with previous studies of patients recovering from depression [31,32]. Further indications of a quadratic response from MCP1 remain to be explored further. However, this is promising preliminary evidence that drumming could modulate similar biological pathways to pharmacological, psychological and other psychosocial interventions for mental health service users.

In our previous study [6], we hypothesized that stress was a key mediator in the improvements in mental health and biological activity found across the drumming intervention. However, no significant changes were found for the Perceived Stress Scale in this study, nor was there a significant decrease in cortisol. These results are in keeping with mindfulness studies for mental health in which changes in cortisol activity have not been consistently demonstrated across longitudinal interventions despite changes in other biomarker levels, including inflammation, being noted [33,34]. Further research needs to be undertaken to explore this finding in this drumming study in more detail, as it is possible that an alternative approach to cortisol sampling, such as taking measurements across the day, would have produced different responses. However, an alternative explanation to be tested alongside this is that, given the strong improvements found in social resilience reported here, the social elements of group drumming had more of an influence on biological response than stress reduction. This would be in keeping with more general theories on the strong social interactions involved in music and the possible biological underpinnings of these [35–37], and the influence of social support on immune function [38–42].

This study was quasi-experimental since, due to the practicalities of recruitment, participants were not randomized. Despite recruiting through the same channels with the same inclusion criteria for both the drumming and control groups, control participants had lower depression scores at baseline than the drumming group. Although all other psychological and demographic measures were matched, a future randomized study would be helpful to confirm results. Mental health service users involved were also from a broad demographic: in keeping with the “community” aim of the drumming intervention, they were of different age groups, ethnicities, had a range of mental health diagnoses, and were at varying stages of treatment (although crucially there were no treatment changes during the course of this study). It would be instructive to focus in future work on specific subgroups, such as those with major depressive disorder or generalized anxiety disorder, to assess where drumming interventions have the greatest therapeutic potential. For future studies, a comparison group either instead of or in addition to a control group would also help elucidate the features of the group drumming intervention responsible for change. Finally, as this was an exploratory analysis of biomarkers, saliva samples were only collected from the drumming group, and aside from excluding participants with self-reported gum disease, we did not directly assess the oral health of participants, and this may have contributed to variations in response between them [19].

In conclusion, this study demonstrates that group drumming can reduce depression and anxiety and improve social resilience in mental health service users over a 6- and 10-week spans. Changes in psychological profiles were found in parallel with reductions in inflammatory response and a shift towards an anti-inflammatory immune profile, in keeping with other successful mental health interventions. The study also demonstrated a longitudinal impact of drumming, both opening new avenues for research and highlighting the practicality and potentially cost-effectiveness of community drumming interventions for mental health patients. Overall, this suggests the therapeutic potential of group drumming interventions with implications for other music-based psychosocial interventions to be explored in future research. Further work will be needed to reveal a more complete picture of the viability of group drumming in clinical practice and ascertain more about the underlying mechanisms.

## Supporting Information

S1 Protocol Study Protocol. (DOCX)

S1 Table Psychological results in the drumming and control groups in weeks 1 (baseline), 6, and 10. (DOCX)

S2 Table Saliva biomarker concentrations in the drumming group in weeks 1 (baseline), 6, and 10. (DOCX)

S1 TREND Checklist Trend Checklist. (PDF)
