# Supplementary material for: Effects of Group Drumming Interventions on Anxiety, Depression, Social Resilience and Inflammatory Immune Response among Mental Health Service Users
Source: PLoS One. 2016 Mar 14;11(3):e0151136. doi: 10.1371/journal.pone.0151136 (PMC4790847; doi:10.1371/journal.pone.0151136)
Supplement: S1 Protocol — (DOCX) [file pone.0151136.s001.docx]

Creative Practice as Mutual Recovery: The impact of group drumming on mental health service users

**Ethics Ref:** 13/LO/1811

| **Study Title:** | Effects of group music interventions for mental health service users on anxiety, depression, social resilience and inflammatory response |
| --- | --- |
| **Chief Investigator:** | Prof Aaron Williamon, Centre for Performance Science, Royal College of Music |
| **Sponsor:** | Royal College of Music |
| **Funder:** | Arts and Humanities Research Council |
|  |  |

**TABLE OF CONTENTS**

[1. SYNOPSIS 4](#_Toc435793541)

[2. BACKGROUND AND RATIONALE 4](#_Toc435793542)

[3. OBJECTIVES AND OUTCOME MEASURES/ENDPOINTS 5](#_Toc435793543)

[4. STUDY DESIGN 6](#_Toc435793544)

[5. Study Participants 6](#_Toc435793545)

[5.1. Inclusion Criteria 6](#_Toc435793546)

[5.2. Exclusion Criteria 6](#_Toc435793547)

[6. STUDY PROCEDURES 7](#_Toc435793548)

[6.1. Recruitment 7](#_Toc435793549)

[6.2. Informed Consent 7](#_Toc435793550)

[6.3. Potential risks and benefits 7](#_Toc435793551)

[6.4. Screening and Eligibility Assessment 7](#_Toc435793552)

[6.5. Baseline Assessments 8](#_Toc435793553)

[6.6. Subsequent Assessment 8](#_Toc435793554)

[6.7. Withdrawal 8](#_Toc435793555)

[6.8. Definition of End of Study 8](#_Toc435793556)

[7. INTERVENTIONS 8](#_Toc435793557)

[8. STATISTICS AND ANALYSIS 8](#_Toc435793558)

[8.1. Description of Statistical Methods 8](#_Toc435793559)

[8.2. The Number of Participants 8](#_Toc435793560)

[8.3. Analysis of Outcome Measures/Endpoints 9](#_Toc435793561)

[9. DATA MANAGEMENT 9](#_Toc435793562)

[9.1. Access to Data 9](#_Toc435793563)

[9.2. Data Recording and Record Keeping 9](#_Toc435793564)

[10. QUALITY ASSURANCE PROCEDURES 9](#_Toc435793565)

[11. ETHICAL AND REGULATORY CONSIDERATIONS 9](#_Toc435793566)

[11.1. Declaration of Helsinki 9](#_Toc435793567)

[11.2. ICH Guidelines for Good Clinical Practice 9](#_Toc435793568)

[11.3. Approvals 9](#_Toc435793569)

[11.4. Reporting 9](#_Toc435793570)

[11.5. Participant Confidentiality 9](#_Toc435793571)

[12. FINANCE AND INSURANCE 10](#_Toc435793572)

[12.1. Funding 10](#_Toc435793573)

[12.2. Insurance 10](#_Toc435793574)

[13. PUBLICATION POLICY 10](#_Toc435793575)

[14. REFERENCES 10](#_Toc435793576)

# SYNOPSIS

| **Study Title** | Effects of group music interventions for mental health service users on anxiety, depression, social resilience and inflammatory response | |
| --- | --- | --- |
| **Internal ref. no. / short title** | 13/LO/1811 | |
| **Study Design** | Quasi-experimental controlled study | |
| **Study Participants** | Adult mental health service users | |
| **Planned Sample Size** | Experimental: 39 to be recruited  Control: 20 to be recruited | |
| **Planned Study Period** | Recruitment from August 2014, study October 2014-December 2014 | |
|  | **Objectives** | **Endpoints** |
| **Primary** | To reduce anxiety and depression | Psychological scale:  Hospital Anxiety and Depression Scale (HADS) |

# BACKGROUND AND RATIONALE

Worldwide, mental health conditions are the leading cause of disability and, along with substance use disorders, are responsible for more of the global health burden than HIV/AIDS, tuberculosis, diabetes, or transport injuries. In the next 20 years, the global lost economic output as a result of mental health conditions will amount to $16 trillion (Whiteford et al., 2013).

Alongside pharmacological and psychotherapeutic treatments for mental health conditions, psychosocial therapies are increasingly appearing, often as an adjunct to conventional treatment as a way of increasing patients’ involvement in their own mental health, encouraging health-promoting behaviours to enhance recovery, and reducing the load of symptoms. A recent systematic review found 69 psychological studies aimed at enhancing mental health and preventing depression or other mental disorders in those at risk or with sub-clinical symptoms (Forsman, Nordmyr, & Wahlbeck, 2011) with interventions including physical exercise, group support, reminiscence or social activities.

One promising psychosocial intervention, which has been recognised as beneficial for mental health for nearly a century is music (Walk, 1928a, 1928b). Indeed, there is now a large body of literature that has demonstrated improved symptoms and reduced severity of conditions, from depression to schizophrenia, in response to music (Erkkilä et al., 2011; Talwar et al., 2006). However, the majority of previous studies have taken place within specific institutions and used a music therapy model, led by a professional music therapist with specific psychological aims. A much less researched area is whether *general* music making within community settings, not led by therapists, can still enhance the mental health and wellbeing of service users. This is an important topic to explore, as a growing number of organisations in the UK and abroad are developing community music interventions for mental health, including Youth Music UK and the Mental Health Foundation. Research into their efficacy is needed to ascertain whether they have a therapeutic effect and to support the design and implementation of future interventions.

One of the community music interventions growing in popularity for mental health is group drumming, perhaps due to the inclusiveness of drumming circles, lack of fine motor skill requirements and strong steadying rhythms. We conducted a preliminary study that explored whether group drumming sessions across six weeks could enhance indicators of mental health (Fancourt et al., In press). Our results showed significant improvements in standardized, self-report measures of depression, social resilience and mental wellbeing among participants. However, this previous study was uncontrolled. Consequently, this planned study seeks to expand on the previous study with a quasi-experimental controlled design.

# OBJECTIVES AND OUTCOME MEASURES/ENDPOINTS

| **Objectives** | **Outcome Measures/Endpoints** |
| --- | --- |
| **Primary Objective** To assess the impact of group drumming on anxiety and depression in mental health service users | Hospital Anxiety and Depression Scale (HADS) |
| **Secondary Objectives**  To assess the impact of group drumming on:  Wellbeing  Social resilience  Psychological stress  Neuroendocrine stress  Inflammatory immune response  To compare whether 6 or 10 weeks is more effective | Warwick-Edinburgh Mental Wellbeing Scale (WEMWBS)  Connor-Davidson Social Resilience Scale (CD-RISC)  Perceived Stress Scale (PSS  Cortisol (through saliva samples)  Cytokines (through saliva samples)  Comparison of results between baseline and week 6 and week 10 |

# STUDY DESIGN

This will be a quasi-experimental controlled study.

# Study Participants

## Inclusion Criteria

Adults of either gender accessing mental health services.

## Exclusion Criteria

The participant may not enter the study if ANY of the following apply:

- Serious mental health problems which might prevent an individual giving informed consent
- Total deafness or severely impaired hearing
- A dementia that would prevent informed consent
- A physical impairment that would prevent participation in the intervention
- A change in care over the course of the 10 weeks of study e.g. commencing a new/different therapy
- A change in medication over the course of the 10 week study
- A new diagnosis over the course of the 10 week study

For saliva samples:

- Use of medication that might affect saliva samples
- Gum disease that might cause local inflammation of the mouth

# STUDY PROCEDURES

## Recruitment

Potential participants will be recruited through advertisements in local borough newsletters (Kensington and Chelsea and Hammersmith and Fulham boroughs), through the Royal College of Music website, through posters in communal spaces such as churches in the local area. Information about the study will also be available in local GP surgeries, mental health day centres and distributed through mental health channels such as online forums. Staff at such locations, including psychologists and psychiatrists, may be able to point service users towards the study. But it is up to the service users themselves to contact us with a view to taking part in the study. We will receive no personal information from health professionals.

## Informed Consent

Participants will personally sign and date the Informed Consent form, co-signed by a member of the research team, before any study activity is performed. Written and verbal versions of the Participant Information sheet will be presented to the participants detailing the exact nature of the study and what it will involve for the participant. It will be clearly stated that the participant is free to withdraw from the study at any time for any reason without prejudice to future care, and with no obligation to give the reason for withdrawal. The participant will be allowed time to consider whether they would like to be involved and the opportunity to ask questions to decide whether they will participate in the study.

## Potential risks and benefits

**Risks:** Participating in participatory drumming workshops involves some degree of physical movement. Participants are able to remain seated throughout, but if a participant becomes tired or finds a particular movement difficult or painful, they can sit out until they feel better. Cushions and a variety of chairs will be available to pick from. We don't anticipate that completion of surveys or provision of saliva samples will be a burden, but if it becomes so, help will be offered from a member of the research team. Participants will have to make their own way to the workshops. To minimise travel inconveniences, participants will mainly be recruited though local networks.

**Benefits:** This research aims to ascertain how musical activities can benefit mental recovery for adults with mental health conditions. So participants will benefit from being able to take part in enjoyable group activities at no cost. These activities are designed to be relaxing and there is no indication from previous studies or current communal activities that group music sessions have any adverse effects on health. The workshops may introduce participants to others who may be able to form a future support network for them; provide them with a sense of group inclusion; teach new musical skills that participants may choose to continue with after the study; and give participants a sense of personal pride in their achievements. As the sessions will be delivered by professional workshop leaders and trained musicians from the Royal College of Music, the standard of music making and teaching

will be very high.

## Screening and Eligibility Assessment

Participants will approach the study team directly if they are interested in taking part in the study. Eligibility criteria will be checked and, if eligible, participants will be sent information sheets and consent forms.

## Baseline Assessments

Data collection at baseline will consist of completing an anonymous questionnaire and providing a saliva sample.

Data collection in questionnaire:

- Hospital Anxiety and Depression Scale (HADS)
- Warwick-Edinburgh mental wellbeing scale (WEMWBS)
- Connor-Davidson Social Provisions Scale (CD-RISC)
- Perceived Stress Scale (PSS)
- Demographic questions

Saliva samples (experimental group only):

- Cortisol
- A range of pro- and anti-inflammatory cytokines (IL4, IL6, IL17, TNFα, MCP1)

## Subsequent Assessment

The data collection carried out in week 1 will be repeated in weeks 6 and 10. For the experimental group, this will also be repeated 3 months following the end of the intervention.

## Withdrawal

If a participant withdraws from the study, data already collected with consent will be retained and used in the study. But no further data will be collected or any other research procedures carried out on or in relation to the participant.

## Definition of End of Study

The end of the study will be once participants have completed all data collection (after 10 weeks for the control group and after 10 weeks + 3 months for the experimental group).

# INTERVENTIONS

Experimental participants will take part in weekly 90-minute group drumming sessions over a period of 10 weeks. They will be split into two groups of 15-20, and sessions will run consecutively on weekday mornings in a hired community space in West London, led by a professional drummer and supported by three students from the Royal College of Music. Sessions will consist of call-and-response exercises and learning drumming patterns that build up into larger pieces. Control participants will be adults who are already taking part in regular community group social activities (e.g. quiz nights, women’s institute meetings and book clubs), but will not take part in any group musical activity during the 10 weeks.

# STATISTICS AND ANALYSIS

## Description of Statistical Methods

Data will be analysed using IBM SPSS Version 22.0.1 (SPSS, Chicago, IL). We will compare results using repeated measures analysis of variance (ANOVAs) with planned simple contrasts, both within and between subjects. Differences with a resultant p value of <.05 will be considered significant. In the event that data is non-parametric, we will use Kruskall-Wallis one-way analysis of variance. However, if biological data is non-parametric, we will attempt to transform prior to analysing parametrically.

## The Number of Participants

Sample size calculations using an alpha of 0.05, power of 0.8 and an effect size of 0.25 (2 groups, 3 timepoints) suggest an overall total of 28 participants will be required (14 per group). For the control group, in order to allow for drop-outs of 30%, 20 participants will be targeted for recruitment. For the experimental group, because of the range of biological markers being tested, we will match sample size with our preliminary study, and so 39 participants will be recruited. Recruitment will be continued until these targets are reached before being closed one week before the drumming starts.

## Analysis of Outcome Measures/Endpoints

Psychological scale scores will be measured and data inputed to IBM SPSS Version 22.0.1. Change from baseline and within groups will be measured. Saliva samples will be shipped to Aeirtec Laboratories and analysed using multiplex ELISAs.

# DATA MANAGEMENT

## Access to Data

Data will only be accessible by the immediate study team. However, direct access will be granted to authorised representatives from the Sponsor or host institution for monitoring and/or audit of the study to ensure compliance with regulations.

## Data Recording and Record Keeping

No patient medical records will be accessed for this study. All data will be stored in linked anonymised form so data is not immediately identifiable. Study data will be stored on secure computers only accessible to the study team. For the three-month follow-up, participants will be sent questionnaires to complete in the post, and this contact information will be stored separately from anonymised data.

# QUALITY ASSURANCE PROCEDURES

The study may be monitored, or audited in accordance with the current approved protocol, ICH GCP, relevant regulations and standard operating procedures.

# ETHICAL AND REGULATORY CONSIDERATIONS

## Declaration of Helsinki

The Investigator will ensure that this study is conducted in accordance with the principles of the Declaration of Helsinki.

## ICH Guidelines for Good Clinical Practice

The Investigator will ensure that this study is conducted in full conformity with relevant regulations and with the ICH Guidelines for Good Clinical Practice (CPMP/ICH/135/95) July 1996.

## Approvals

The protocol, informed consent form, participant information sheet and any proposed advertising material will be submitted to the Head of the Centre for Performance Science at the Royal College of Music and the NHS Research Ethics Committee (REC) for written approval. The Investigator will submit and, where necessary, obtain approval from the above parties for all substantial amendments to the original approved documents.

## Reporting

An End of Study notification and final report will be submitted to the same parties and data uploaded to clinicaltrials.gov.

## Participant Confidentiality

Patient data will remain anonymous to the study team throughout the study and there will be no access to patient medical records. Participants will be assigned unique identification codes. All documents will be stored securely and only accessible by study staff and authorised personnel.

# FINANCE AND INSURANCE

## Funding

The study is being funded by the Arts and Humanities Research Council

## Insurance

Royal College of Music has an indemnity and insurance policy that will cover the entire study.

# PUBLICATION POLICY

The Investigators will be involved in reviewing drafts of the manuscripts, abstracts, press releases and any other publications arising from the study. Authors will acknowledge that the study was funded by the Arts and Humanities Research Council. Authorship will be determined in accordance with the ICMJE guidelines and the Vancouver Protocol and other contributors will be acknowledged.

# REFERENCES

Erkkilä, J., Punkanen, M., Fachner, J., Ala-Ruona, E., Pöntiö, I., Tervaniemi, M., … Gold, C. (2011). Individual music therapy for depression: randomised controlled trial. *The British Journal of Psychiatry*, *199*(2), 132–139. http://doi.org/10.1192/bjp.bp.110.085431

Fancourt, D., Perkins, R., Ascenso, S., Atkins, L., Kilfeather, S., Carvalho, L. A., … Williamon, A. (In press). Group drumming modulates cytokine activity in mental health service users: a preliminary study. *Psychotherapy and Psychosomatics*.

Forsman, A. K., Nordmyr, J., & Wahlbeck, K. (2011). Psychosocial interventions for the promotion of mental health and the prevention of depression among older adults. *Health Promotion International*, *26*(suppl 1), i85–i107. http://doi.org/10.1093/heapro/dar074

Talwar, N., Crawford, M. J., Maratos, A., Nur, U., McDERMOTT, O., & Procter, S. (2006). Music therapy for in-patients with schizophrenia. *The British Journal of Psychiatry*, *189*(5), 405–409. http://doi.org/10.1192/bjp.bp.105.015073

Walk, A. (1928a). Music in Correctional Institutions. The Musician’s Contribution to Modern Mental Treatment. Music as a Means of Discipline. A Systematic Music Program for Mental Hospitals. Educational Features of an Institutional Music Program. *The British Journal of Psychiatry*, *74*(306), 521–522. http://doi.org/10.1192/bjp.74.306.521-a

Walk, A. (1928b). The Utilization of Music in Prisons and Mental Hospitals. *The British Journal of Psychiatry*, *74*(306), 521–521. http://doi.org/10.1192/bjp.74.306.521

Whiteford, H. A., Degenhardt, L., Rehm, J., Baxter, A. J., Ferrari, A. J., Erskine, H. E., … Vos, T. (2013). Global burden of disease attributable to mental and substance use disorders: findings from the Global Burden of Disease Study 2010. *The Lancet*, *382*(9904), 1575–1586. http://doi.org/10.1016/S0140-6736(13)61611-6
