# Supplementary material for: Effects of Group Drumming Interventions on Anxiety, Depression, Social Resilience and Inflammatory Immune Response among Mental Health Service Users
Source: PLoS One. 2016 Mar 14;11(3):e0151136. doi: 10.1371/journal.pone.0151136 (PMC4790847; doi:10.1371/journal.pone.0151136)
Supplement: S1 Table — (DOCX) [file pone.0151136.s002.docx]

**S1 Table. Psychological data.** Psychological results in the drumming and control groups in weeks 1 (baseline), 6, and 10.

| Scale | Baseline | | Week 6 | | Week 10 | |
| --- | --- | --- | --- | --- | --- | --- |
| Mean ± SEM | **Drumming** | **Control** | **Drumming** | **Control** | **Drumming** | **Control** |
| HADSA | 11.03 ± 0.83 | 9.93 ± 1.16 | 10.07 ± 0.77 | 9.20 ± 1.07 | 8.83 ± 0.70 | 9.60 ± 0.97 |
| HADSD | 8.90 ± 0.79 | 4.27 ± 1.10 | 6.76 ± 0.73 | 4.80 ± 1.02 | 5.48 ± 0.62 | 4.73 ± 0.87 |
| CDRISC | 46.93 ± 3.47 | 57.85 ± 4.83 | 54.62 ± 3.18 | 57.40 ± 4.42 | 57.52 ± 3.16 | 59.07 ± 4.39 |
| WEMWBS | 39.61 ± 1.91 | 44.67 ± 2.61 | 42.68 ± 1.94 | 46.47 ± 2.65 | 45.75 ± 1.80 | 47.00 ± 2.46 |
| PSS | 23.17 ± 1.28 | 21.87 ± 1.78 | 22.45 ± 1.34 | 18.07± 1.86 | 19.52 ± 1.12 | 16.00 ± 1.55 |
