# Supplementary material for: Effects of Group Drumming Interventions on Anxiety, Depression, Social Resilience and Inflammatory Immune Response among Mental Health Service Users
Source: PLoS One. 2016 Mar 14;11(3):e0151136. doi: 10.1371/journal.pone.0151136 (PMC4790847; doi:10.1371/journal.pone.0151136)
Supplement: S2 Table — (DOCX) [file pone.0151136.s003.docx]

**S2 Table. Biological data.** Saliva biomarker concentrations in the drumming group in weeks 1 (baseline), 6, and 10.

| Biomarker | Baseline | Week 6 | Week 10 |
| --- | --- | --- | --- |
| Mean ± SEM (ng/ml) |  |  |  |
| Cortisol | 3.40 ± 0.15 | 3.37 ± 0.13 | 3.24 ± 0.16 |
| Mean ± SEM (pg/ml) |  |  |  |
| IL17 | 0.80 ± 0.03 | 0.90 ± 0.06 | 0.85 ± 0.04 |
| IL4 | 1.62 ± 0.10 | 1.77 ± 0.10 | 1.83 ± 0.10 |
| IL6 | 1.50 ± 0.10 | 1.60 ± 0.10 | 1.52 ± 0.10 |
| MCP1 | 4.35 ± 0.15 | 3.92 ± 0.19 | 4.27 ± 0.17 |
| TNFα | 1.17 ± 0.08 | 1.22 ± 0.09 | 1.17 ± 0.10 |
